# Curcumin Inhibits Apoptosis of Chondrocytes through Activation ERK1/2 Signaling Pathways Induced Autophagy

**DOI:** 10.3390/nu9040414

**Published:** 2017-04-21

**Authors:** Xiaodong Li, Kai Feng, Jiang Li, Degang Yu, Qiming Fan, Tingting Tang, Xiao Yao, Xiaoqing Wang

**Affiliations:** 1 Department of Orthopedic Surgery, Shanghai Ninth People’s Hospital, Shanghai Jiaotong University School of Medicine, Shanghai 200011, China; lucas021@sina.com (X.L.); jsycfk@126.com (K.F.); shdlijiang8901@163.com (J.L.); ydg163@126.com (D.Y.); chillow@163.com (Q.F.); tingtingtang@hotmail.com (T.T.); 2 Shanghai Key Laboratory of Orthopedic Implants, Department of Orthopedic Surgery, Shanghai Ninth People’s Hospital, Shanghai Jiaotong University School of Medicine, Shanghai 200011, China; 3 Shanghai Zhangjiang Puhui Institute of Translational Medicine, Shanghai 200128, China; sdmu923@hotmail.com; 4 Present address: No. 639, Zhizaoju Road, Huangpu District, Shanghai 200011, China

**Keywords:** osteoarthritis, curcumin, apoptosis, autophagy, ERK

## Abstract

Osteoarthritis (OA) is an inflammatory disease of load-bearing synovial joints that is currently treated with drugs that exhibit numerous side effects and are only temporarily effective in treating pain, the main symptom of the disease. Consequently, there is an acute need for novel, safe, and more effective chemotherapeutic agents for the treatment of osteoarthritis and related arthritic diseases. Curcumin, the principal curcuminoid and the most active component in turmeric, is a biologically active phytochemical. Evidence from several recent in vitro studies suggests that curcumin may exert a chondroprotective effect through actions such as anti-inflammatory, anti-oxidative stress, and anti-catabolic activity that are critical for mitigating OA disease pathogenesis and symptoms. In the present study, we investigated the protective mechanisms of curcumin on interleukin 1β (IL-1β)-stimulated primary chondrocytes in vitro. The treatment of interleukin (IL)-1β significantly reduces the cell viability of chondrocytes in dose and time dependent manners. Co-treatment of curcumin with IL-1β significantly decreased the growth inhibition. We observed that curcumin inhibited IL-1β-induced apoptosis and caspase-3 activation in chondrocytes. Curcumin can increase the expression of phosphorylated extracellular signal-regulated kinases 1/2 (ERK1/2), autophagy marker light chain 3 (LC3)-II, and Beclin-1 in chondrocytes. The expression of autophagy markers could be decreased when the chondrocytes were incubated with ERK1/2 inhibitor U0126. Our results suggest that curcumin suppresses apoptosis and inflammatory signaling through its actions on the ERK1/2-induced autophagy in chondrocytes. We propose that curcumin should be explored further for the prophylactic treatment of osteoarthritis in humans and companion animals.

## 1. Introduction

Osteoarthritis (OA) is a progressive and degenerative disease and is a leading cause of pain and disability that affects millions of people worldwide. It is characterized by the degradation of articular cartilage, the modification of the subchondral bone, and the inflammation of the synovial membrane [1]. Despite the high prevalence of OA, there is currently no cure or effective treatment that halts or reverses disease progression. The conventional treatments for OA concentrate on the control of symptoms (i.e., pain) and function mostly by the use of acetaminophen or non-steroidal anti-inflammatory drugs (NSAIDs) [2,3,4]. However, these drugs sometimes cause serious gastrointestinal and cardiovascular adverse events, especially with long-term use. Thus, there is clear and urgent need for food or food-derived products, the so-called nutraceuticals [5,6], that provide effective and safe treatment for OA.

**Figure 1:**
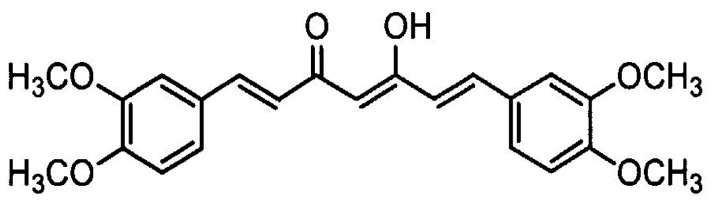
Chemical structures of curcumin. Curcumin is derived from the rhizomes of turmeric (*Curcuma longa*).

Curcumin is a polyphenol extracted from turmeric (Figure 1), which has been safely used in foods such as curries for a long time [7]. Curcumin is a promising therapeutic food material because of its anti-inflammatory and antioxidative functions, and has long been used as an anti-inflammatory treatment in traditional Chinese and Ayurvedic medicine [7]. Curcumin has been expected to be effective for a range of diseases related to chronic inflammation, including cancer, cardiovascular disease, metabolic syndrome, Alzheimer disease, osteoarthritis, and other common diseases and aging conditions [7,8,9,10,11]. Furthermore, it has been reported that curcumin can be a potent inhibitor of the production of inflammatory and catabolic mediators by chondrocytes [12]. Since OA and related osteoarticular conditions of synovial joints are characterized by inflammation, a better understanding of the biochemistry of curcumin and its biological actions in joint tissues may facilitate the development of clinically safe, orally administered therapeutic agents for treating joint diseases.

Pro-inflammatory cytokines such as interleukin 1β (IL-1β) plays important roles in the pathogenesis of osteoarthritis (OA). Once IL-1β is released, it stimulates the synthesis of more pro-inflammatory cytokines, which also induce chondrocyte apoptosis [13]. In addition, these cytokines are potent stimulators for the de novo production of catabolic enzymes such as matrix metalloproteinases (MMPs), which are responsible for excessive cartilage matrix degradation in OA [14,15]. IL-1β also suppresses the expression of cartilage-specific extracellular matrix components including collagen type II and cartilage-specific proteoglycans [16]. The catabolic changes can accelerate the degeneration of cartilage. In this study, we investigated whether curcumin can inhibit the apoptosis-promoting effects of IL-1β.

Autophagy, which is known as a conserved catabolic process, has been widely studied in human degenerative diseases [17,18]. Autophagy mediates the degradation of damaged proteins and dysfunctional organelles for energy recycling to maintain the metabolic homeostasis of the cell under certain stress [19]. Several studies have reported that autophagy might have a protective effect in the degenerative process of the chondrocytes or fibro-chondrocytes [20,21,22]. The decrease in autophagy with age may be as one of the reasons for OA and activation of autophagy may reduce the severity of OA [23]. However, the effects of curcumin on autophagy are still unknown in the chondrocytes.

Growing evidence supports the idea that signaling pathway malfunctions in chondrocytes and synovial cells are involved in aging and joint diseases such as OA and rheumatoid arthritis (RA). A study by Shakibaei et al. showed that extracellular signal-regulated kinases 1/2 (ERK1/2) is related to chondrocyte apoptosis [24]. Treatment of OA with novel agents that can simultaneously target multiple cellular signaling pathways in chondrocytes will benefit from effectively downregulating inflammation without adverse systemic effects. One study has shown that curcumin can activate Mitogen-activated protein kinase (MEK)/ extracellular signal-regulated kinase (ERK) signaling, a pathway that is involved in the maintenance of chondrocyte differentiation and survival [24]. However, it is not clear whether there is a relationship between autophagy and ERK signaling pathway in chondrocytes after curcumin or IL-1β treatment.

Therefore, the aim of the present study was to examine if there is a link between curcumin and autophagy on IL-1β-stimulated primary chondrocytes in vitro, and if so, what signaling pathway is involved.

## 2. Materials and Methods

### 2.1. Animals

Male Sprague-Dawley rats (200–220 g) were purchased from Sino-British Sippr/BK Lab Animal Ltd., Shanghai, China. The rats were housed under laminar flow in an isolated room with controlled temperature and at a 12/12 (light/dark) schedule. Food and water were available ad libitum. All experimental procedures were ethically approved by the Animal Use and Care Committee of Shanghai Jiaotong University (ethic code: 2012279) and were conducted in accordance with the National Institute for Health “Guide for the Care and Use of Laboratory Animals”.

### 2.2. Reagents

Growth medium Dulbecco's Modified Eagle Medium F-12 (DMEM/F12) and fetal bovine serum (FBS) were obtained from Hyclone (Logan, UT, USA); Curcumin, rapamycin, collagenase-II, and bovine serum albumin (BSA) were purchased from Sigma-Aldrich (St. Louis, MO, USA); 3-methyladenine (3-MA) was purchased from Gene Operation (Ann Arbor, MI, USA) and prepared as a stock solution of 100 mM in phosphate buffered saline (PBS); IL-1β was from Invitrogen (Carlsbad, CA, USA); Terminal deoxynucleotidyl transferase dUTP nick end labeling (TUNEL) Apoptosis Assay was from BD PharMingen (West Grove, CA, USA).The following monoclone antibodies were obtained from Cell Signaling Technology (Danvers, MA, USA): anti-beclin-1, anti-LC3, anti-Bcl-2, anti-cleaved caspase-3, anti-ERK, anti-phosphorylated ERK, and anti- Glyceraldehyde 3-phosphate dehydrogenase (GAPDH). Anti-collagen II antibody was purchased from Sigma-Aldrich (St. Louis, MO, USA); Tetramethylrhodamine (TRITC)-conjugated secondary antibody was obtained from Boster Biological Engineering, Wuhan, China; Phalloidin and 4′,6-diamidino-2-phenylindole (DAPI) were purchased from Yeasen, Shanghai, China and KeyGEN Biotech, Nanjing, China respectively; Cell-counting kit-8 assay (CCK-8) was obtained from DOJINDO molecular technologies (Kumamoto, Japan)

### 2.3. Chondrocytes Isolation, Culture, and Identification

Cartilage tissue samples from healthy femoral head articular cartilage obtained from 4-week-old SD rats were dissociated enzymatically in 0.25% trypsin/ ethylenediaminetetraacetic acid (EDTA) and 0.2% collagenase-II at 37 °C and 5% CO_2_ for 6 h. Primary chondrocytes were plated in high-density monolayers (1.0 × 10^5^ cells/cm^2^) in Petri dishes and cultured in Ham’s F-12/Dulbecco’s modified Eagle’s medium (50/50) containing 10% fetal bovine serum, 25 mg/mL ascorbic acid, 50 IU/mL streptomycin, 50 IU/mL penicillin, 2.5 mg/mL amphotericin B, essential amino acids, and l-glutamine. All the cells used in the experiment were freshly obtained primary chondrocytes.

Immunofluorescence staining was used to identify chondrocytes. Cells were seeded in chamber slides. Chondrocytes were washed with PBS twice at 37 °C, fixed with 4% paraformaldehyde for 20 min, permeabilized with 0.5% Triton X-100 buffer (Beyotime, Jiangsu, China) for 5 min at room temperature, and blocked with 1% bovine serum albumin for 10 min at 4 °C. The cells were incubated with anti-collagen II antibody for 1 h, washed three times for 5 min each, and then incubated with TRITC-conjugated secondary antibody for 1 h at room temperature. Cytoskeleton and nuclei were counter-stained with Phalloidin and DAPI for 10 min. Fluorescent images were observed and analyzed using Olympus DP version software (Olympus Corporation, Tokyo, Japan).

### 2.4. Cell Viability and Proliferation Assay

**Figure 2:**
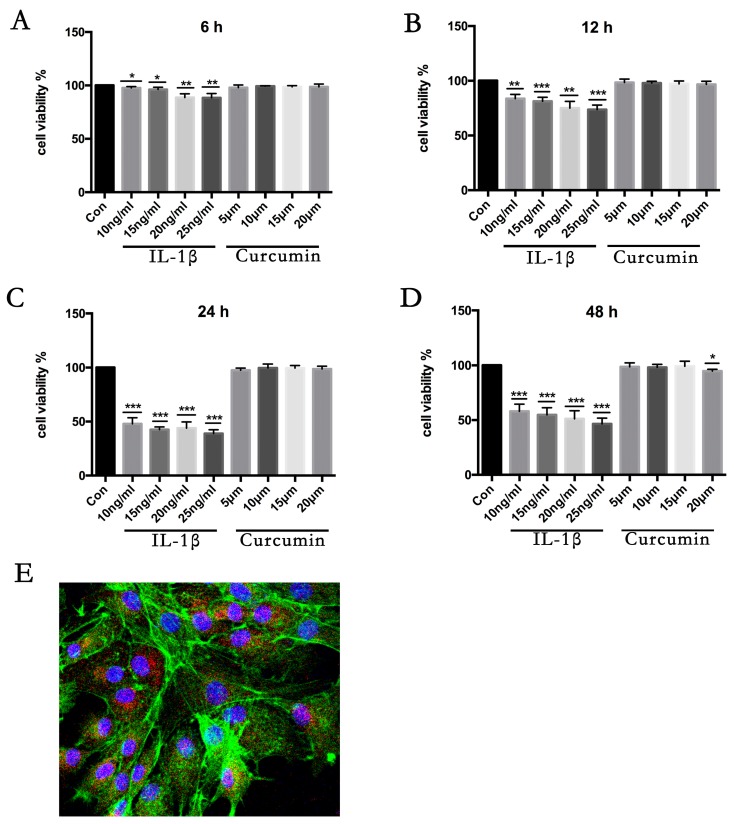
Effects of curcumin and interleukin 1β (IL-1β) on the viability and proliferation of primary chondrocytes in vitro. To evaluate the effect of curcumin or IL-1β-induced cytotoxicity, primary chondrocytes were treated with 10 ng/mL, 15 ng/mL, 20 ng/mL, and 25 ng/mL IL-1β or 5 μM/L, 10 μM/L, 15 μM/L, and 20 μM/L curcumin for the following times: 6 h (**A**), 12 h (**B**), 24 h (**C**), and 48 h (**D**). IL-1β has a significant cytotoxic effect on chondrocytes. Results are expressed as means ± SD for experiments performed in triplicate. * *p* < 0.05, ** *p* < 0.01, and *** *p* < 0.001 compared with controls. (**E**) Immunofluorescence identification of collagen II (red) in primary chondrocytes.

Collagen II immunofluorescence staining results showed that all the cells in our experiment are chondrocytes (Figure 2E). Cell viability was assessed by cell-counting kit-8 assay (CCK-8). Briefly, 5 × 10^3^ chondrocytes per well were cultured for 24 h in a 96-well-plate and then treated with 10 ng/mL IL-1β alone for the indicated time periods, or pretreated with 10 μM curcumin or 7.5 μM rapamycin for 4 h followed by co-treatment with 10 ng/mL IL-1β and 10 μM curcumin or 7.5 μM rapamycin for the indicated time periods. From this 100 mM stock, experimental low concentrations of curcumin (5 μM, 10 μM, 15 μM, and 20 μM) were prepared in DMEM/F-12 and added to the appropriate wells. Similarly, the concentrations of IL-1β (10 ng/mL, 15 ng/mL, 20 ng/mL, and 25 ng/mL) were prepared in DMEM/F-12 from the stock concentration of 0.1 mg/mL. A Dimethyl Sulfoxide (DMSO) control containing a volume equivalent to that found in the highest curcumin concentration was included on each plate to ensure that any observed effects were not due to the carrier solvent. Cytotoxicity was assessed after 12 h, 24 h, and 48 h. The absorbance of each well was read on a spectrophotometer (Thermo) at 450 nm (A450). Three independent experiments were performed in quintuplicate. The percentage viability was calculated by using the formula % Cell viability = {(OD of treated) (OD of control)} × 100.

### 2.5. TUNEL Staining

For apoptotic cells, DNA fragmentation was detected by TUNEL assay (Roche Applied Science, Pennsburg, Germany). For the TUNEL assay, monolayer chondrocytes in 96-well plate were treated with corresponding reagents and cultured at 37 °C. Cells were fixed with 4% paraformaldehyde (PFA) for 20 min at ambient temperature (AT), rinsed three times with PBS, permeabilized with 0.1% Triton-X 100 in 0.1% sodium citrate for 2 min on ice, and then rinsed three times with PBS. Apoptotic chondrocytes were stained using the situ cell death detection kit AP used for TUNEL staining in in vitro evaluations. Then chondrocyte nuclei were counterstained with DAPI. Thereafter, coverslips were mounted onto glass slides which were observed under confocal microscope in order to view the chondrocyte apoptosis. The percentage of apoptotic cells was calculated as the number of fluorescein labeled cells per DAPI-stained nucleus. The number of cells with morphological features of apoptotic cell death was determined by scoring 100 cells from 20 different microscopic fields.

### 2.6. MDC Assay

Monodansylcadaverine (MDC) was combined with autophagy-lysosome in cytoplasm, emitting fluorescence. Chondrocytes were seeded on sterile glass slides in cell culture media. Primary chondrocytes were either pretreated or not with 10 μM curcumin or 7.5 μM rapamycin for about 2 h, following treatment with 10 ng/mL IL-1β and 10 mM 3-MA for the indicated time periods. Then the chondrocytes were incubated with MDC (0.05 mM) for 30 min at 37 °C. Following incubation, chondrocytes were washed three times with PBS, sucking the glass slide dry. Then coverslips were mounted onto the glass slides which were then immediately viewed by confocal microscopy. Excitation wavelengths were 360–380 nm and Olympus DP version software (Olympus Corporation, Tokyo, Japan) was used. The number of cells with morphological features of autophagy was determined by scoring 100 cells from 20 different microscopic fields.

### 2.7. Transmission Electron Microscopy (TEM)

Cells were fixed for 1 h with Karnovsky-fixative followed by post-fixation in 1% OsO4 solution (0.1 M phosphate buffer). Monolayer cell pellets were rinsed and dehydrated in an ascending alcohol series before being embedded in Epon and cut on a Reichert-Jung Ultracut E (Reichert-Jung, Darmstadt, Germany). Ultrathin sections were contrasted with 2% uranyl acetate/lead citrate. A transmission electron microscope (TEM 10, Zeiss, Jena, Germany) was used to examine the cultures.

### 2.8. Western Blot Analysis

To determine the effect of curcumin on IL-1β-induced apoptosis, primary chondrocytes were lysed in radioimmunoprecipitation assay (RIPA) buffer supplemented with protease inhibitor cocktail (Roche, Stockholm, Sweden), and cell debris was removed by centrifugation. The total protein concentration of whole cell, nuclear, and cytoplasmic extracts (30 μg) was determined using the bicinchoninic acid (BCA) Protein Assay Kit (Thermo; Waltham, MA, USA) using BSA as a standard. Equal quantities (30 μg protein per lane) of total proteins were separated by sodium dodecyl sulfate polyacrylamide gel electrophoresis (SDS-PAGE) (10%, 12.5%, 15% gels).

Thereafter, proteins were blotted onto polyvinylidene fluoride (PVDF) membranes and blocked for 1 h with 2.5% skimmed milk at ambient temperature. The membranes were incubated with primary antibodies against LC3, Beclin-1, Bcl-2, Cleaved Caspase 3, ERK, p-ERK, and GAPDH overnight under 4 °C. Membranes were washed three times with TBST, and were incubated with secondary antibodies for one hour. They were finally washed three times in Tris-buffered saline with Tween (TBST). The density (specific binding) of each band was measured by using the Odyssey infrared imaging system (Li-Cor; Lincoln, NE, USA).

### 2.9. Flow Cytometry (FCM) Assay 

Following the respective treatment as above, the cells were washed with ice-cold PBS and trypsinized without EDTA. Removing the supernatant after centrifugation, the cells (1 × 10^5^) were suspended in 200 μL binding buffer, and incubated with 4 μL Annexin V-FITC for 10 min at ambient temperature free from light. Then 4 μL PI and 300 μL binding buffer were added into the flow tube. The rate of apoptosis (%) was measured by a flow cytometer (Epics Altra II; Beckman Coulter Inc., Brea, CA, USA).

### 2.10. Statistical Analysis

All the experiments were repeated three to four times. Data is presented as mean values ± SE. Statistical analysis was done by unpaired Student’s *t* test for two groups and one-way ANOVA for more than two groups using SPSS 13.0 software (SPSS Inc., Chicago, IL, USA). *p* values < 0.05 were considered significant. The symbols *, **, and *** denote *p* < 0.05, *p* < 0.01, and *p* < 0.001, respectively.

## 3. Results

### 3.1. Cell Viability Assay

The effect of curcumin on cell survival is shown in Figure 2. The cytotoxicity of the curcumin was measured by CCK-8 assay. As shown in Figure 2, the curcumin exerted no significant cytotoxic effect at concentrations up to 20 μM at different time points. Compared to the negative control group (0.2% DMSO), cells treated with IL-1β (10 ng/mL) showed significantly reduced cell viability after 6 h. These results suggested that the IL-1β treatment significantly reduced the cell viability of chondrocytes in dose and time dependent manner.

### 3.2. Curcumin Suppresses IL-1β Induced Chondrocyte Apoptosis

The effect of curcumin on IL-1β-induced apoptosis in chondrocytes was examined by TUNEL staining and flow cytometry (FCM) assay. Serum starved primary isolated chondrocytes were either treated with 10 ng/mL IL-1β for 24 h or pretreated with 10 μM curcumin for 4 h and co-treated with 10 ng/mL IL-1β and 10 μM curcumin for the same time periods. The apoptosis induced by IL-1β was characterized by FCM and TUNEL analysis. The TUNEL procedure stains nuclei that contain nicked DNA, a characteristic exhibited by cells in the early stages of apoptotic cell death. 

**Figure 3:**
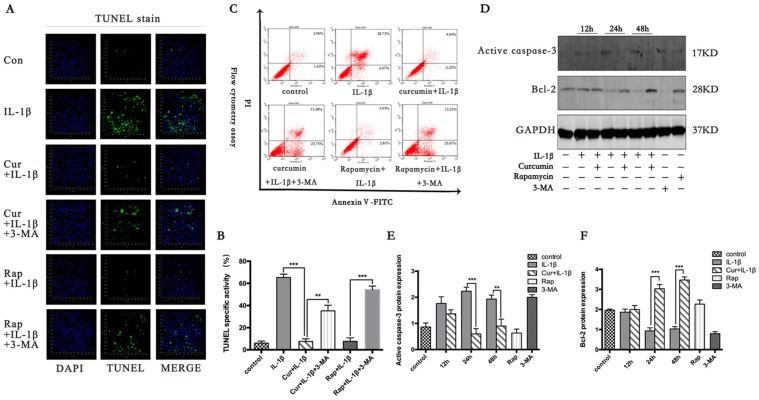
Effects of curcumin on IL-1β-induced apoptosis in primary chondrocytes. (**A**) Apoptotic cells were visualized using terminal deoxynucleotidyl transferase mediated dUTP nick end labeling (TUNEL) fluorescence immunocytochemistry (green). Nuclei were counterstained with 4′,6-diamidino-2-phenylindole (DAPI) (blue); (**C**) Flow cytometric detection of apoptosis in the chondrocytes. Apoptotic cells labeled with Annexin V and propidium iodide (PI) fluorescence were estimated by flow cytometry. The percentages of cells in each quadrant is indicative of: upper left, necrotic cells; lower left, live cells; lower right, early apoptotic cells; and upper right, late apoptotic cells; (**D**) Accumulation of cleaved caspase 3 and bcl-2 was visualized by western blot. Results shown in (**B**,**E**,**F**) are expressed as means ± SD for experiments performed in triplicate. * *p* < 0.05, ** *p* < 0.01, and *** *p* < 0.001.Con, control; Rap, rapamycin; Cur, curcumin; 3-MA, 3-Methyladenine.

As shown in Figure 3A–C, the cells in the control group were negative for TUNEL staining. When chondrocytes were treated solely with IL-1β, the number of apoptosis chondrocytes was significantly higher than the groups that had been pretreated with curcumin.

To further investigate the interaction between apoptosis and autophagy, we measured the apoptosis level after the autophagy inducer and inhibitor treatments (Figure 3A–C). The chondrocytes were pretreated with rapamycin (10 μM, Sigma-Aldrich, St. Louis, MO, USA) or 3-MA (10 nM, Sigma-Aldrich, St. Louis, MO, USA) for 2 h before treatment with curcumin or IL-1β. Rapamycin, the autophagy inducers, significantly decreased the apoptotic level induced by IL-1β (*p* < 0.001). In contrast, the apoptotic cells significantly increased after treatment with curcumin when autophagy was suppressed by 3-MA (*p* < 0.001). Taken together, the results suggest that curcumin could protect the chondrocytes from IL-1β-induced apoptosis through autophagy.

### 3.3. Curcumin Suppresses the Apoptotic Pathway Mediated by Bcl-2

To characterize the mechanism of curcumin protecting chondrocytes from apoptosis induced by IL-1β, the protein level of Bcl-2 and cleaved caspase-3 were analyzed by western blot (Figure 3D–F). Western blot analysis revealed that the expression level of Bcl-2 from 24 h to 48 h was declining in groups which were treated with 10 ng/mL IL-1β compared with the groups that were pretreated with curcumin at the same time points (*p* < 0.001). The expression trend of active caspase 3 was contrary to Bcl-2. 

### 3.4. Curcumin Induces Autophagy in Chondrocytes

**Figure 4:**
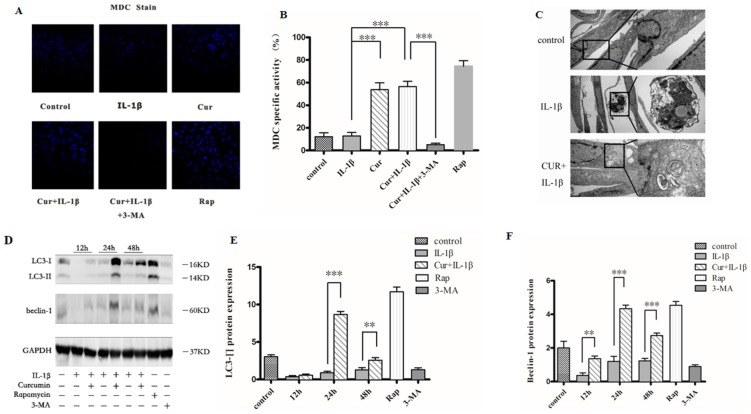
Curcumin activated autophagy within chondrocytes. The curcumin induced autophagy was detected by monodansylcadaverine (MDC) staining ((**A**), quantified in (**B**)) and transmission electron microscope (TEM) (**C**) at 24 h. Accumulation of autophagosomes was observed in chondrocytes that were pretreated with curcumin (**C**). Accumulation of beclin1 and light chain 3 (LC3)-II upon autophagy activation was visualized by western blot ((**D**), quantified in (**E**) and (**F**)). Results in (**B**,**E**,**F**) are expressed as means ± SD for experiments performed in triplicate. * *p* < 0.05, ** *p* < 0.01, and *** *p* < 0.001. Cur, curcumin; Rap, rapamycin; 3-MA, 3-Methyladenine.

To determine whether curcumin can induce autophagy in chondrocytes, MDC staining, transmission electron microscope (TEM, and western blot analysis were used. MDC accumulates in mature autophagic vacuoles (AVs), such as autophagolysosomes, but not in the early endosome compartments [25]. MDC staining can be used to detect autophagic vacuoles. When cells are viewed with a fluorescence microscope, AVs stained by MDC appear as distinct dot-like structures distributed within the cytoplasm or localized in the perinuclear regions. As shown in Figure 4A, there was an increase in the number of MDC labeled vesicles at 24 h after curcumin treatment, which was also the case for the rapamycin treatment. The results indicate an induction of AV formation by curcumin. The effects of curcumin can be blocked by autophagy inhibitor 3-MA. 3-MA inhibits autophagy via its transient suppressive effect on class III PI3K. Class III PI3-kinase is essential for autophagy initiation. Moreover, TEM results showed that apoptosis bodies appeared in the chondrocytes treated with IL-1β for 24 h, while pretreatment with curcumin increased autophagosomes in chondrocytes and decreased the apoptosis bodies (Figure 4C). In accordance with the MDC staining results, the western blot results showed that pretreatment with curcumin can significantly increase LC3-Π and beclin-1 protein levels (Figure 4D). All the data suggested that curcumin increased autophagic activity in chondrocytes.

### 3.5. Curcumin Involves the Activation of MAPK/ERK1/2 Signaling Pathway

**Figure 5:**
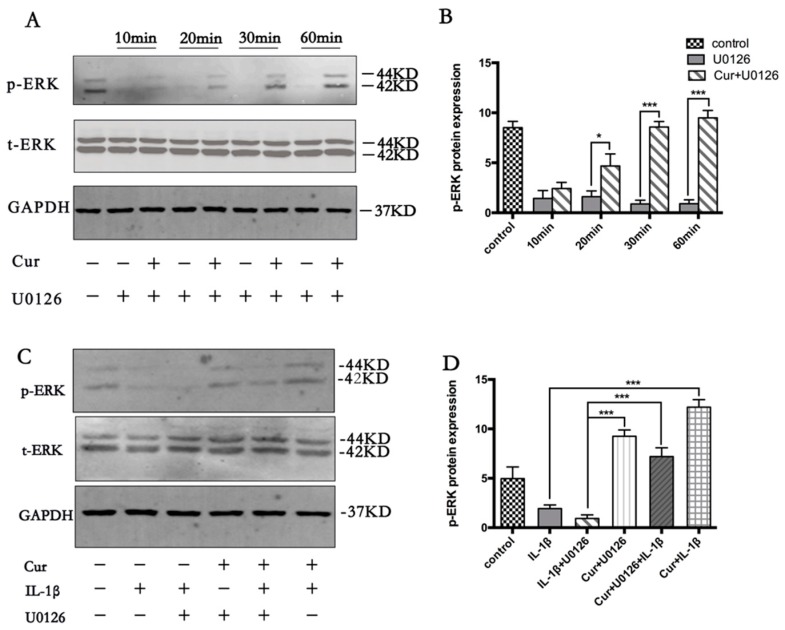
Curcumin reverses U0126-induced inhibition of extracellular signal-regulated kinases 1/2(ERK1/2) in primary chondrocytes. (**A**) p-ERK was visualized by western blot in U0126-treated chondrocytes. Serum-starved chondrocytes were preincubated with 10 μm curcumin for indicated time points and co-treated with 10 μm U0126 for 10 min, 20 min, 30 min, and 60 min. Curcumin pretreatment reversed U0126-induced ERK1/2 inhibition in a time-dependent manner. The pan ERK1/2 was not affected (and quantified in (**B**)). (**C**) Effects of curcumin on IL-1β-induced inhibition of mitogen-activated protein kinase (MAPK)/ERK1/2 pathway in primary chondrocytes in vitro. Serum-starved chondrocytes were pre-stimulated with 10 μm curcumin alone for 4 h and then co-treated with IL-1β (10 ng/mL) and/or 10 μm U0126 for 24 h. Some cultures were left untreated and evaluated after 24 h. Results in (**B**,**D**) are expressed as means ± SD for experiments performed in triplicate. * *p* < 0.05, ** *p* < 0.01, and *** *p* < 0.001. Cur, curcumin.

U0126 suppresses MEK1/2, which is a kinase for ERK1/2. To examine whether curcumin blocks the U0126-induced inhibition of ERK1/2, serum-starved chondrocytes were pretreated with 10 μM curcumin for the indicated time followed by 1 μM U0126 stimulation for 30 min. As shown in Figure 5A,B, curcumin stimulated U0126-induced ERK1/2 inhibition in a time-dependent manner.

It is known that mitogen-activated protein kinase (MAPK)/ERK1/2 signaling pathway regulates cell apoptosis and proliferation. To evaluate whether curcumin can modulate the expression of ERK1/2, we examined U0126-stimulated primary chondrocytes with or without pretreatment of curcumin by western blot analysis at different time points (Figure 5A,B). U0126 notably inhibits the phosphorylation of ERK1/2. In contrast to this, the treatment of curcumin stimulated the phosphorylation of ERK1/2 in a time-dependent manner in chondrocytes (Figure 5A,B).

Furthermore, we also investigated whether the protective effect of curcumin against IL-1β was related with phosphorylation of ERK1/2. Primary chondrocytes were incubated with IL-1β (10 ng/mL) or U0126 (10 μM) alone for the indicated time or were preincubated with curcumin (10 μM) for 2 h and then co-treated with IL-1β (10 ng/mL) or U0126 (10 μM) for the indicated time. As shown in Figure 5C,D, pretreatment with curcumin significantly upregulated the level of p-ERK1/2 in co-treated IL-1β or U0126-stimulated cultures compared with primary chondrocytes stimulated with IL-1β and U0126 alone (*p* < 0.001).

### 3.6. Curcumin Induces Autophagy through Activation ERK1/2 Signaling Pathways

**Figure 6:**
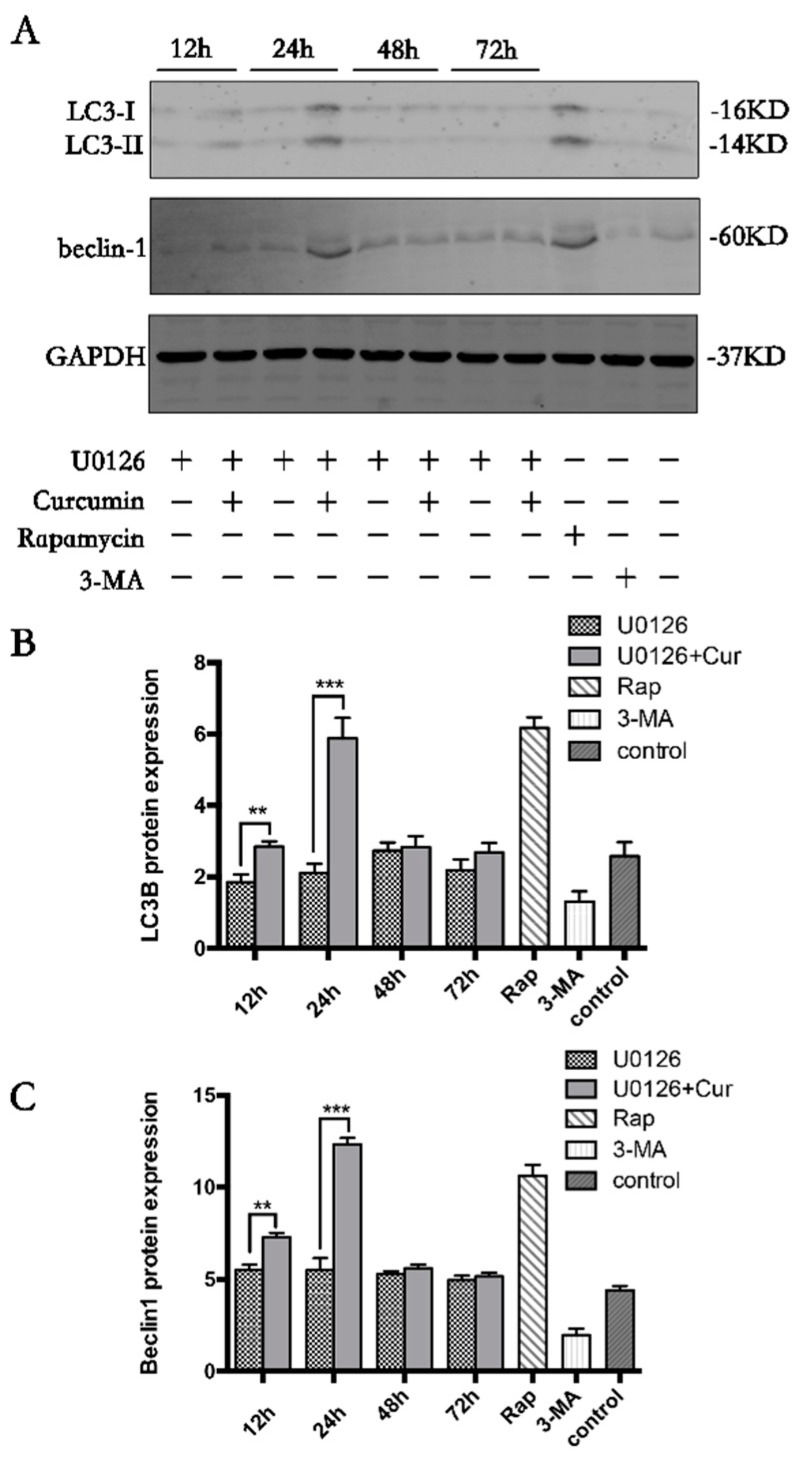
Curcumin could activate autophagy for U0126-induced inhibition of ERK1/2 in primary chondrocytes. Accumulation of beclin-1 and LC3-II upon autophagy activation was visualized by western blot ((**A**), quantified in (**B**) and (**C**)). Results in (**B**,**C**) are expressed as means ± SD for experiments performed in triplicate.* *p* < 0.05, ** *p* < 0.01, and *** *p* < 0.001. Rap, rapamycin; 3-MA, 3-Methyladenine.

We next speculated that inhibition of MAPK/ERK1/2 might affect the expression of autophagy marker LC3-II and beclin-1. Therefore, we treated primary chondrocytes with U0126 (10 μM) alone for the indicated time periods, or pretreated with 10 μM curcumin, and established that curcumin was able to release the inhibition of MAPK/ERK1/2 signaling pathway and induce the expression of light chain 3 (LC3)-II and beclin-1 in a time-dependent manner, especially at 24 h (Figure 6, *p* < 0.001), suggesting that MAPK/ERK1/2 signaling pathway activity was necessary for inducing autophagy.

## 4. Discussion

The results of this study present following findings: (1) treatment of chondrocytes with 10 ng/mL IL-1β results in morphological alterations and apoptosis, with apoptotic bodies observed under TEM and through FCM; (2) The IL-1β-induced chondrocytes apoptosis was abolished through pretreatment with curcumin in a time-dependent manner; (3) The mechanism of curcumin anti-apoptotic effects was the induction of chondrocyte autophagy, and this correlated with activation of the MAPK/ERK1/2 signaling pathway; (4) Curcumin suppressed U0126-induced downregulation of the p-ERK1/2 in a time-dependent manner and IL-1β or U0126 inhibition of activation and phosphorylation of ERK1/2 could be clearly blocked by curcumin.

Here we show that autophagy is required for inhibiting chondrocyte apoptosis. Curcumin is known for its potent anti-inflammatory and anti-oxidant properties, which could alleviate the process of osteoarthritis [24,26,27]. There are some previous studies that have shown that autophagy is constitutively active in chondrocytes [21,28] and our studies confirmed this.

The appropriate autophagy activation plays an important role in maintaining normal chondrocyte metabolism. Autophagy plays a housekeeping role in removing misfolded or aggregated proteins, and clearing damaged organelles; as such, it has a key role in preventing diseases such as cancer, neurodegeneration, cardiomyopathy, diabetes, liver diseases, autoimmune diseases, and infections. Autophagy is a well-conserved mechanism and has been confirmed to be important in various physical events [29,30,31,32]. Among the human autophagy genes, beclin-1 and LC3 are major regulators and markers of the autophagy pathway [33]. Beclin-1 could form a complex with type III phosphatidylinositol that allows nucleation of the autophagic vesicles. LC3-I is converted into LC3-II, which is then attached to the membrane of the autophagosome during the process of autophagy activation. The BH3 domain of beclin-1 interacts with anti-apoptotic protein Bcl-2, which inhibits the beclin-1 induced activation of autophagy.

Articular cartilage appears to be highly susceptible to the accumulation of aging-related changes, in part due to the relatively low turnover of extracellular matrix and cells. Therefore, as the only cells in articular cartilage, chondrocyte death is associated with the breakdown of cartilage [34]. The moderate autophagy could effectively inhibit cell apoptosis [21]. In our present study, an increase in autophagic activity induced by short-term curcumin treatment was detected and showed a time-dependent manner, where the autophagy activation was at its peak at 24 h, before declining gradually. The above results suggest that short-term treatment with curcumin might also activate autophagy. Autophagy is likely to be a self-protective process in chondrocytes induced by curcumin in response to IL-1β stimulation. In order to confirm this, we adopted autophagy activator rapamycin and autophagy inhibitor 3-MA. When chondrocytes were pretreated with curcumin, then co-treated with IL-1β and 3-MA, chondrocyte apoptosis clearly increased and the autophagy of the chondrocyte was inhibited. Western blot analysis confirmed that the pathological apoptosis marker cleaved caspase-3 was upregulated by IL-1β stimulation, while its expression was downregulated after curcumin treatment. Bcl-2, as the member of the Bcl family, has an anti-apoptosis effect. From 24 h to 48 h, the expression of Bcl-2 was reduced in chondrocytes that were incubated with IL-1β, while pretreatment with curcumin was able to reverse the expression of Bcl-2. Studies have demonstrated that cytochrome c may participate in the activation of caspase 3 and that Bcl-2 blocks the release of cytochrome c from mitochondria.

The mitogen-activated protein kinase (MAPK) pathway is stimulated and important for chondrocyte differentiation and survival. Apoptosis of human chondrocytes in vitro is induced by specific inhibition of the MAPK [35]. As a branch of MAPK, the ERK signaling pathway can inhibit the apoptotic pathway in different cell types [36]. In this study, we examined the essential role of the ERK pathway on chondrocyte autophagy by treating chondrocytes with U0126. The markers of autophagy were decreased in groups where chondrocytes were treated solely with U0126 in comparison to those that were pretreated with curcumin, suggesting that a functional death-mediating signaling cascade is associated with chondrocyte autophagy. Previous research has proved that inhibition of the ERK1/2 signaling pathway induces apoptosis of chondrocytes [35], which is consistent with the results of our study, where autophagy and apoptosis have an antagonistic relationship.

**Figure 7:**
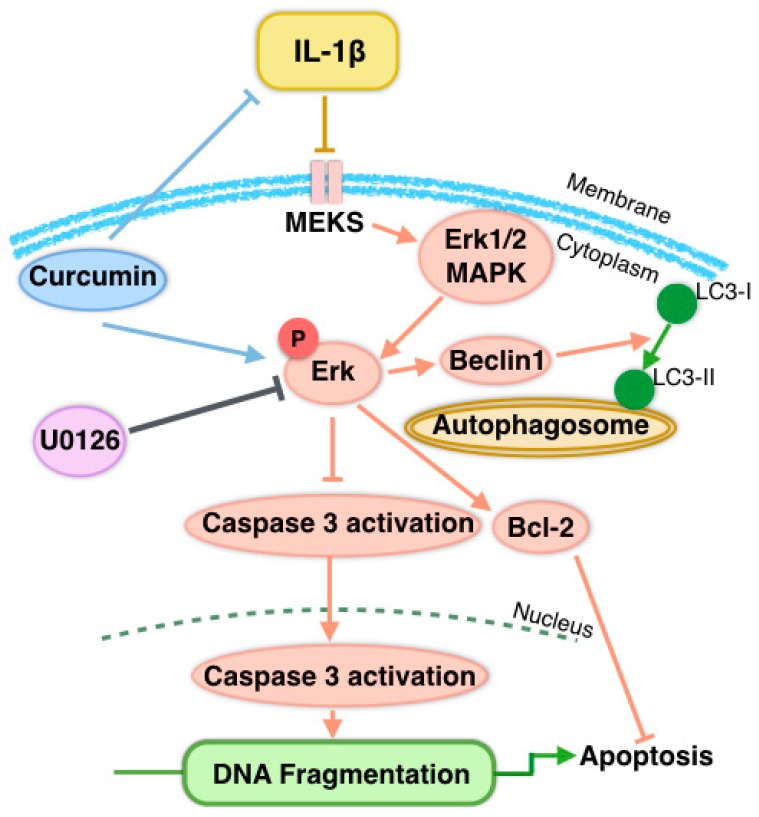
Curcumin induced autophagy via the ERK1/2 signal pathway to protect chondrocytes from apoptosis. IL-1β stimulates the IL-1β receptor, initiating an intracellular signal transduction cascade, which inhibits the cytoplasmic MAPK/ERK1/2 signaling pathway, then activates proinflammatory and pro-apoptotic gene production. ERK1/ERK2, a downstream kinase of the MAPK pathway, regulates the expression and activity of various transcription factors. Specific inhibition of ERK1/ERK2 by U0126 or IL-1β results in cleavage of caspase-3 in primary chondrocytes in vitro. Since activation of caspase-3 and DNA fragmentation are common features of apoptosis, the specific inhibition of the Ras-mitogen-activated kinase leads to chondrocyte apoptosis. Curcumin blocks the inhibition effect of U0126 or IL-1β on the MAPK pathway and activates autophagy in chondrocytes. Beclin-1 promotes LC3 activation. Recruitment and integration of LC3B-II into the growing phagophore, eventually results in the formation of autophagosomes.

Altogether, our data suggest that autophagy is important for chondrocyte survival, and inhibition of this process leads to caspase-dependent death of chondrocytes, while the self-activation of autophagy is a protective mechanism against apoptosis under short-term treatment with curcumin within 48 h. This suggests that the anti-inflammatory and anti-apoptotic effects of curcumin are mediated, at least in part, through the MAPK/ERK1/2 signaling transduction pathway (Figure 7). This provides theoretical guidance for the clinical treatment of OA.

## 5. Conclusions

In conclusion, our study revealed that curcumin leads to increased autophagy in chondrocytes and that autophagy is apro-survival mechanism under IL-1β stimulation. Curcumin enhances autophagy and attenuates IL-1β-mediated apoptosis at least in part via ERK1/2 activation-dependent autophagy. Thus, we propose that enhancing autophagy through increased phosphorylated ERK1/2 expression could be a potential therapeutic strategy in OA. Although ongoing laboratory research will shed more light on the biochemical action of curcumin, randomized double-blind and placebo controlled clinical trials are necessary to confirm our in vitro findings.
